# Primary osteosarcoma of the breast: A case report

**DOI:** 10.3892/ol.2014.1981

**Published:** 2014-03-19

**Authors:** MARIUSZ SZAJEWSKI, WIESŁAW JANUSZ KRUSZEWSKI, MACIEJ CIESIELSKI, URSZULA ŚMIAŁEK-KUSOWSKA, MAKSYMILIAN CZEREPKO, JAROSŁAW SZEFEL

**Affiliations:** 1 Department of Surgical Oncology, Gdynia Oncology Centre, PCK’s Maritime Hospital in Gdynia, 81-519 Gdynia, Poland; 2 Department of Propedeutics of Oncology, Faculty of Health Sciences, Medical University of Gdańsk, 81-519 Gdynia, Poland; 3 Department of Anatomical Pathology, PCK’s Maritime Hospital in Gdynia, 81-519 Gdynia, Poland

**Keywords:** osteosarcoma, breast, tumour

## Abstract

Osteosarcoma (OS) located in the breast is an extremely rare, malignant neoplasm. The current study presents the diagnostic process, treatment and follow-up of a 67-year-old female with OS of the breast. The diagnosis was made according to diagnostic imaging methods and microscopic examination with additional immunohistochemical staining. As a surgical treatment, tumourectomy with intraoperative histological examination and simple breast amputation was performed. No adjuvant chemotherapy was administered following surgery. Lung and bone metastases occurred without local recurrence 14 months following the surgery. In the present study, the details of the diagnostic examinations performed are evaluated and the consecutive phases of diagnostic and therapeutic proceedings are examined in comparison with the knowledge in the literature.

## Introduction

Extraskeletal osteosarcomas (OSs) are extremely rare, malignant neoplasms. In the general population, primary osteosarcomas constitute <1% of all malignant neoplasms of the breast. OS of the breast may constitute between 12.5 to 17% of all breast sarcomas (2,3). In the literature, single cases of OS have been reported to be located in the thyroid, kidneys, urinary bladder, colon, heart, testes, gall bladder and cerebellum (4). Primary osteogenic sarcoma is an extremely aggressive neoplasm. It is characterised by a high percentage of early, local recurrences and high metastatic potential, mostly spreading via the blood. Metastases are most commonly observed in the lungs and bones (1,5,6). The current study presents the details of the diagnostic process, treatment and 18-month follow-up of a 67-year-old patient with primary OS of the left breast. Written informed consent was obtained from the patient’s family.

## Case presentation

A 67-year-old female patient was admitted to the Oncology Clinic of the Gdynia Oncology Centre (GCO; Gdynia, Poland) with a self-detected lump in the left breast. Medical history, physical examination and additional diagnostic tests, including mammography, ultrasonography (USG) of the breast, core-needle biopsy of the palpable breast abnormality, radiological imaging of chest and laboratory tests, were performed. According to the test results and the clinical evaluation, a preliminary diagnosis of OS of the left breast was established.

**Figure 1:**
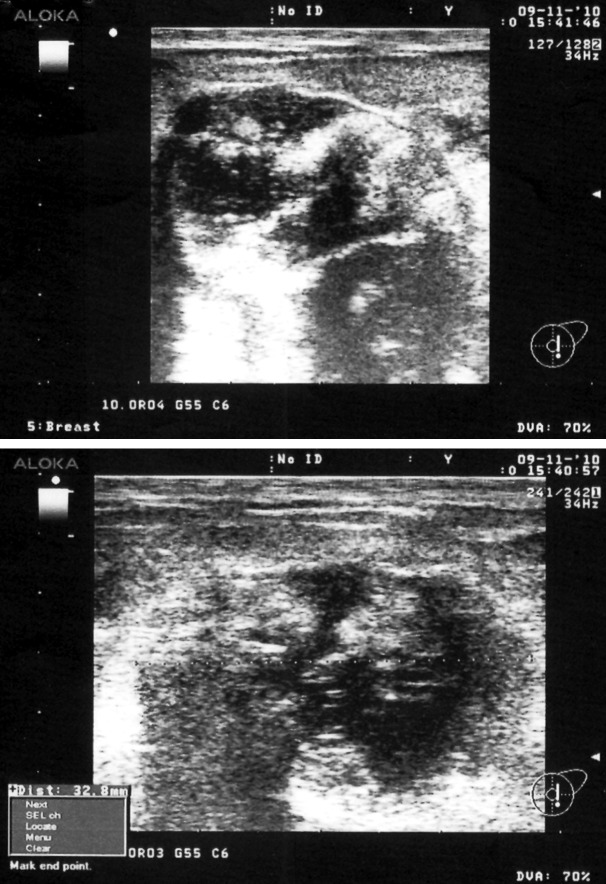
Ultrasound image of primary breast osteosarcoma.

The mammogram revealed a 5-cm, smooth-contoured lesion of a regular density in the left breast, located behind the nipple. On the mammography images of the left breast obtained 5 years earlier, there was no such lesion present, nor were any other pathological lesions. No lesion was demonstrated in the right breast. Additional USG of the breast revealed a polycyclic, oval lesion with heterogeneous echogenicity behind the nipple (Fig. 1). No axillary lymphadenopathy was observed. Medical imaging indicated a malignant neoplasm of the left breast. A thorax radiograph and laboratory test results did not reveal any abnormalities. Fine-needle biopsy demonstrated a high density, amorphous protein mass with diffused neutrophils and shadows of necrotic cells. According to the results of the core biopsy, there was suspicion of primary extraskeletal OS or a malignant phyllodes tumour.

One month from the beginning of the diagnostic procedures, the patient was admitted to the Oncological Surgery Ward of GCO. Besides tumour of the breast, the patient did not report any other discomforts. The patient had previously given birth twice at the ages of 22 and 23 years. The patient breast-fed her children for 12 and 3 months, respectively and did not experience any mastitis or mechanical injuries of the breast area. The patient never received any hormonal therapy medicines, radiotherapy or treatment for any breast lesion. Several years earlier, the patient underwent surgery for varicose veins of the lower extremities, and has suffered from hypoacusis for several years. The mother of the patient was diagnosed with breast cancer at 33 years. Upon physical examination, a palpable tumour of 7 cm in diameter was located behind the nipple of the left breast of the patient, clinically staged at cT3N0. The lesion did not cause any pain and there was no leakage from the nipple. Besides hypoacusis, no other abnormalities were observed.

During the surgery, tumour resection with normal tissue margins was achieved, confirmed by an intra-operative histological examination. The frozen section examination led to a diagnosis of malignant non-epithelial OS. A decision to extend the surgical margins for a simple mastectomy of the left breast was made. No complications occurred during the surgery and the patient was discharged after 2 days. A decision regarding further treatment was postponed until a full histopathological report was obtained.

**Figure 2:**
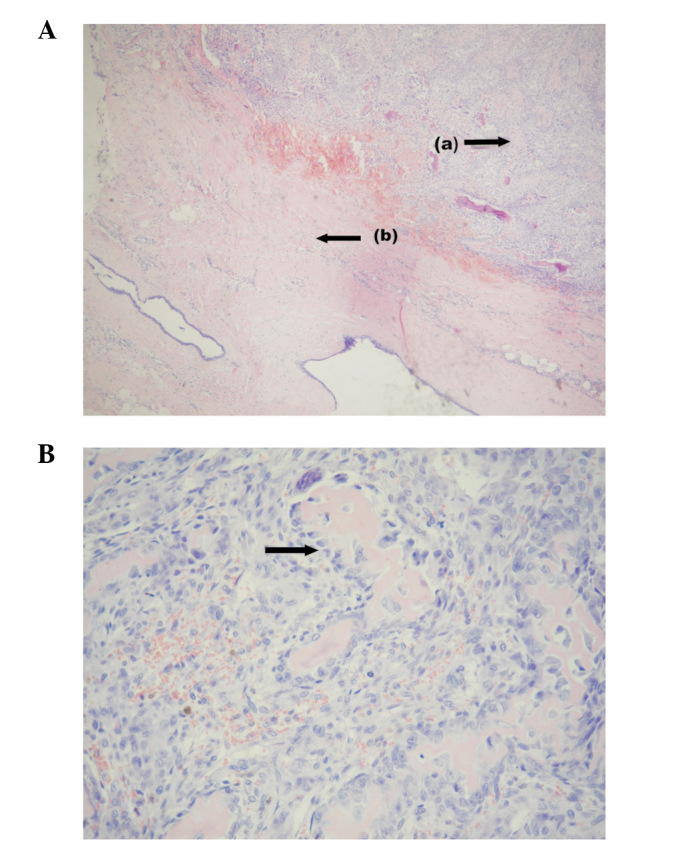
(A) Photomicrograph showing breast osteosarcoma (OS): The tumour tissue with (a) osteoid foci and (b) mammary gland tissue (magnification, ×40). (B) Primary breast OS; the arrow indicates osteoblasts surrounding an osteoid (magnification, ×200).

The histopathological report described a solid, polycyclic, well-circumscribed tumour that was 6 cm in diameter. Macroscopically, the tumour tissue was grey-pink, hyaline, with nodules of decay and fibrosis. Microscopic examination showed the presence of a neoplastic osteoid. Immunohistochemical staining did not reveal any epithelial component [cytokeratin (CK) AE1/AE3(-), CK7(-) and epithelial membrane antigen(-)], however, vimentin immunoreactivity was detected. The mitotic index [Ki-67(+), >70%]indicated high mitotic activity of the tumour. The diagnosis of extraskeletal OS was confirmed (Fig. 2A and B). No other tumour nodules were present in the amputated breast tissue. Surgical margins were 3 cm in distance from the tumour site in all directions. Follow-up visits every 3 months were recommended to the patient.

**Figure 3:**
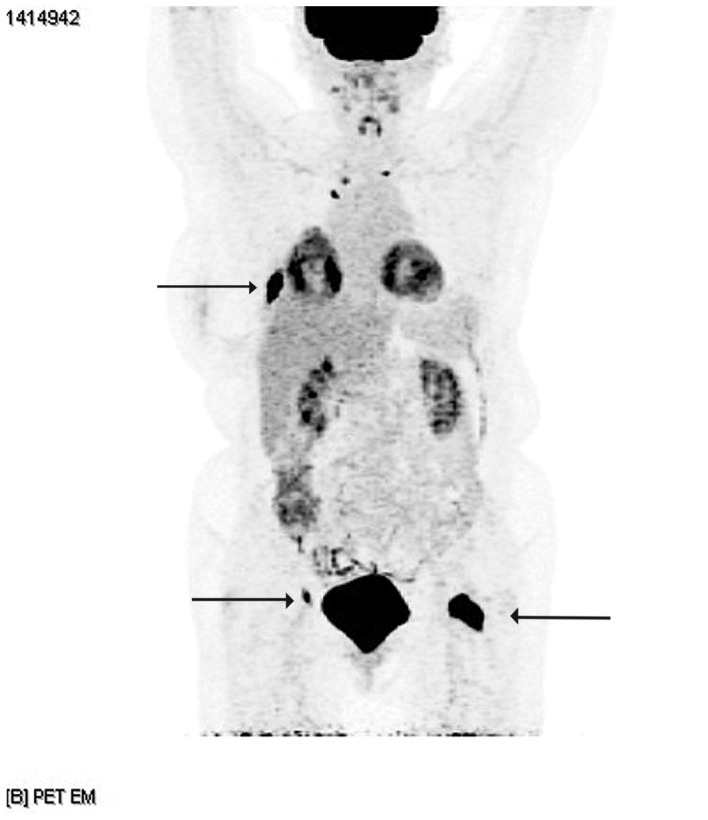
Whole-body positron emission tomography-computed tomography of the bones. The arrows indicate metastatic nodules of primary breast osteosarcoma.

During the first year of follow-up, no evidence of relapse was detected and 14 months following the diagnosis, a thorax radiograph showed metastatic nodules in the lower site of the right lung. A thorax computed tomography (CT) scan confirmed this observation. Due to bone pain, a positron emission tomography-CT scan was performed, which demonstrated tumour metastases in the bones (thoracic section of the spine, rib VIII on the right side, right ischial bone and collum of the thigh bone) (Fig. 3). Additionally, a pathological break of the collum of the right thigh bone was diagnosed. Besides palliative radiotherapy for the collum of the thigh bone, no other oncological treatment was administered. The patient succumbed to the disease due to dissemination 18 months following surgery.

## Discussion

The mechanism of tumourigenesis of primary breast OS is unclear. It has been indicated that the tumours arise from totipotent mesenchymal cells of the breast stroma. Another possible mechanism is transformation from a pre-existing breast fibroadenoma or phyllodes tumour (2,6,7). Radiotherapy may induce the formation of breast sarcomas, predominantly angiosarcomas and chest wall OS. Such an association was observed in patients treated with radiotherapy for epithelial breast neoplasms (8–10). In the reported case, there was no data indicating histogenesis of OS from a pre-existing fibroadenoma or phyllodes tumour; lack of previous breast pathology and a negative result in mammography 2 years earlier. An association with radiotherapy was also excluded.

As reported in the literature, primary breast OS mainly occurs in females of >60 years, which is consistent with the observation in the present case (1,5–7,11–14). The malignancy has also been observed in younger females (1,4,8,15,16). A report of male primary breast OS also exists (1). Similar to the current patient, a physical examination revealed a palpable, firm, mobile mass that did not cause contraction of the nipple. Usually, it is without bloody leakage from the nipple and axillary lymph node enlargement (1,5–8,11,13–15). The presence of palpable enlarged axillary lymph nodes on the side of the tumour has only been reported in two primary breast OS cases (4,12). Similar to the radiology examinations of the current patient, in mammography imaging, OS presents as a well-circumscribed, oval and firm calcified mass (6–8,11–15). In USG imaging, the tumour may be observed with blurred outlines, heterogeneous echogenicity and focal calcification (8,11,14,15).

Histopathological evaluation is fundamental for the diagnosis of primary extraskeletal OS. According to the criteria established in the study by Allan and Soule (17), the basis of the diagnosis of extraskeletal OS should be as follows: Presence of neoplastic osteoid or bone tumor in the microscopic section, origination from the bones excluded and absence of an epithelial component. In the present case study, these criteria are fulfilled.

The fundamental element of the treatment of primary breast OS is surgery, which consists of complete tumour resection with normal tissue margins or a simple mastectomy (1,5,6,13). Metastases in lymph nodes in breast OS cases are uncommon, as is OS localised in the bones (1,7,8,15,16,18). A lymphadenectomy in patients with OS is justified in situations where axillary lymphadenopathy is observed. In such cases, palpable, enlarged lymph nodes may be the site of neoplastic metastases (4,12). In the present case, the simple mastectomy was performed as no enlarged lymph nodes were observed during the physical examination.

To obtain an increased survival time in cases of primary breast OS of the bones, multi-agent chemotherapy, including doxorubicin, cisplatin, high-dose methotrexate with leucovorin and ifosfamide, is used (18). Applying this option is not standard treatment for the management of breast OS patients due to the limited number of clinical trials where polychemotherapy has been used in cases of this extremely rare tumour (1,4–6,11–15). In a situation where tumour-free surgical margins cannot be obtained, postoperative radiotherapy is sometimes advisable (4,5,8,19–20).

Due to the rarity of the described tumour, results are absent regarding overall survival in primary breast OS. In one study, the 5-year survival rate was evaluated as <38%. In the group of 50 patients with primary breast OS, metastases were observed in 41%, mainly in the lung, and in almost half of these, metastases were detected >1 year post-diagnosis. The patients with detected metastases succumbed ≤20 months from diagnosis (median, 2 months) (1).

In the current study, a case of primary breast OS, a rare, malignant tumour of the breast is presented. The follow-up confirms that local therapy with assurance of adequate tumour-free margins may effectively protect a patient against relapse, even in the situation of a large tumour. Systemic dissemination of breast OS remains the greatest problem in treatment.
